# Introducing a Quality-Driven Approach for Federated Learning

**DOI:** 10.3390/s25103083

**Published:** 2025-05-13

**Authors:** Muhammad Usman, Mario Luca Bernardi, Marta Cimitile

**Affiliations:** 1 Department of Engineering, University of Sannio, 82100 Benevento, Italy; bernardi@unisannio.it; 2 Department of Law and Digital Society, Unitelma Sapienza University, 00161 Rome, Italy; marta.cimitile@unitelmasapienza.it

**Keywords:** federated learning, machine learning, data fusion, data imbalance, neural network

## Abstract

The advancement of pervasive systems has made distributed real-world data across multiple devices increasingly valuable for training machine learning models. Traditional centralized learning approaches face limitations such as data security concerns and computational constraints. Federated learning (FL) provides privacy benefits but is hindered by challenges like data heterogeneity (Non-IID distributions) and noise heterogeneity (mislabeling and inconsistencies in local datasets), which degrade model performance. This paper proposes a model-agnostic, quality-driven approach, called DQFed, for training machine learning models across distributed and diverse client datasets while preserving data privacy. The DQFed framework demonstrates improvements in accuracy and reliability over existing FL frameworks. By effectively addressing class imbalance and noise heterogeneity, DQFed offers a robust and versatile solution for federated learning applications in diverse fields.

## 1. Introduction

The rapid growth of pervasive systems has made distributed real-world data across various devices increasingly valuable for training machine learning models [1,2]. Traditional centralized learning methods are widely used across sectors such as healthcare, finance, retail, and transportation, where data originate from multiple and diverse sources [3]. However, these methods face significant limitations, particularly in storage and computational capacity, as well as security and privacy concerns when large numbers of devices are involved [4,5,6]. To address these challenges, distributed data parallelism has been proposed, where multiple machines train a model on different data subsets in parallel [7]. While this approach improves scalability and resource utilization, it does not fully resolve the privacy and security issues inherent in centralized data sharing.

Federated learning (FL) offers an alternative decentralized framework, allowing machine learning models to be trained on distributed data across nodes or clients without requiring data to leave the local device [8,9]. This approach preserves data privacy and security during training, making it especially suitable for sensitive domains [10,11,12]. FL has demonstrated promising results in various applications [13], yet it is not without challenges. Two key issues, data heterogeneity and noise heterogeneity, can significantly compromise the quality and performance of FL models [14].

Data heterogeneity arises from non-independent and identically distributed (Non-IID) data across different clients [15]. Local data distributions often vary significantly, leading to imbalances where minority classes are harder to learn compared to majority classes. This imbalance can degrade the performance of the global model as highlighted in recent studies [16,17]. Noise heterogeneity, on the other hand, refers to the presence of mislabeled or inconsistent data across clients, a common issue in FL due to the varied quality of local datasets [18]. Together, these challenges make developing a robust FL framework a critical task for researchers and practitioners alike [18].

Existing solutions for data heterogeneity, such as iterative model averaging [19,20], have shown some success in handling Non-IID data. However, these approaches do not adequately address the problem of noise heterogeneity, leaving room for further improvement.

This paper introduces DQFed, a novel approach designed to tackle both data and noise heterogeneity in federated learning. By addressing these challenges, DQFed enhances model performance and reliability in decentralized settings.

To mitigate class imbalance, DQFed evaluates the degree of imbalance at each client and assigns weights to their contributions during aggregation. Clients with more pronounced class imbalances are given higher influence on the global model, ensuring that minority classes are better represented.

Similarly, to address noise heterogeneity, DQFed uses a semi-supervised variational autoencoder to identify mislabeled data in local datasets. During model fusion, a weighted approach based on the so-called Noise Reduction Penalty (NRP) metric is applied to downweight the contributions of clients with higher levels of mislabeled data, thereby minimizing their adverse impact on the global model.

Finally, DQFed integrates a robust aggregation algorithm that combines weighted contributions from all clients. This comprehensive approach ensures a more accurate and reliable federated learning model, even in the presence of heterogeneous and noisy data. Experimental evaluations demonstrate that DQFed outperforms existing FL baselines, providing a strong foundation for robust and scalable decentralized machine learning.

The empirical validation aims to answer the following questions:

- **RQ1.** To what extent is DQFed robust in handling class imbalance?
- **RQ2.** To what extent is DQFed robust in addressing mislabeled data?
- **RQ3.** How does the robustness of DQFed change with an increasing number of clients?
- **RQ4.** To what extent is DQFed more effective compared to state-of-the-art (SOA) approaches?

The empirical evaluation of the proposed DQFed method is conducted on 25 datasets derived from the widely recognized and freely available CIFAR-10 dataset (https://www.cs.toronto.edu/~kriz/cifar.html) accessed on 10 January 2024. We also perform an ablation-style study on RQ1 and RQ2 to discuss the individual impacts of imbalance and noise. Although Convolutional Neural Networks (CNNs) are used as a case study in this experimentation to validate the effectiveness of DQFed, it is important to emphasize that the proposed approach is inherently model-agnostic. The strategies employed in DQFed, such as weighted aggregation, noise handling, and class imbalance management, are designed to work independently of the specific architecture of the underlying model. This flexibility allows DQFed to be applied across a wide range of machine learning models, including Recurrent Neural Networks (RNNs), Transformers, and other architectures, as long as the aggregation requirements are met.

However, an essential requirement for the aggregation process is that the models being trained on clients must be homologous. This means that while the type of model can vary across use cases or domains, all participating clients must train the same type of model with identical architectures. This is because the aggregation of model updates—whether through parameter averaging or weighted aggregation—requires compatibility in the structure of the model parameters being combined. The model-agnostic nature of DQFed lies in its focus on addressing challenges intrinsic to federated learning, such as class imbalance and noise heterogeneity, rather than being tied to specific model architectures.

The remainder of this paper is organized as follows: Section 2 reviews related work, providing an overview of existing approaches and their limitations. Section 3 introduces the background concepts necessary for understanding the proposed framework. Section 4 presents a detailed description of the methodology employed in this study, while Section 5 outlines the experimental setup. The results and their discussion are provided in Section 6, highlighting key findings and implications. Finally, Section 7 concludes the paper, summarizing the contributions and suggesting directions for future research.

## 2. Related Work

This section reviews the literature related to our work, focusing on imbalance and mislabeling detection and resolution in federated learning models.

### 2.1. Unbalancing in Federated Learning

Despite the promising accuracy of FL in various settings, its performance tends to be more variable in the case of Non-IID data. Optimizing machine learning models for Non-IID data has been a critical challenge in recent years, given the widespread prevalence of such data in real-world scenarios [21]. Non-IID data can take various forms, including covariate shift, prior probability shift, concept drift, and imbalance. In this study, we focus primarily on imbalanced data, as they pose the most critical challenge within the context of FL. Therefore, throughout this paper, the term Non-IID will first refer to data with high levels of class imbalance. The authors in [22] conclude with the support of a mathematical analysis that Non-IID data significantly reduce FL model accuracy. Starting from this consideration, the survey proposed in [23] describes the various types of class imbalance in FL systems and provides an overview of the existing techniques for addressing class imbalance data. Further exploration of how various Non-IID factors affect FL accuracy can be found in [17]. According to [23] in FL, several ML techniques have been developed to address class imbalance. These can be categorized into three main approaches: sampling-based techniques, algorithm-centered techniques, and system-centered techniques. The first is to adjust the class distribution by preprocessing the training data. Some solutions are described in [24].

Algorithm-centered techniques [25,26] modify the learning algorithm to give more focus to minority classes. Finally, system-centered techniques [27] address the issue within the FL framework using aggregation, personalization, system modifications, and meta-learning.

System center techniques are further categorized into aggregation methods, personalization of clients, system modifications, and meta-learning as discussed in [23]. In aggregation-based methods, the model aggregation process can be improved by weighting local models based on evaluation metrics, not just data volume. This can help address the class imbalance in federated learning. When employing personalized federated learning, individual clients can prioritize their specific data during model training. This approach allows for the creation of customized models that are better suited to their unique needs. The system modification approach focuses on changing the architecture of federated learning settings as discussed in [28]. Meta-learning approaches as referenced in [12] aim to improve the system’s ability to learn effectively, particularly for minority classes.

The approach proposed in this study can be considered an example of a system-centered technique since it performs a sort of system modification by introducing a penalty for nodes that show higher imbalance. Moreover, the survey [23] also discusses the limitations of the existing approaches by highlighting that several techniques are applied exclusively at the local level. In this way, they never consider that clients may have conflicted patterns. According to this, it will be useful to propose methods that introduce a balance between the global model, and the local models. The proposed approach allows for balancing the contribution of local nodes by introducing the weights.

### 2.2. Mislabeling in Federated Learning

Several studies have addressed the issue of mislabeling in the federated learning setting. Some methods consider all clients all together, unlike methods that treat clients individually. For example, Fed-DR-Filter, introduced in [29], is a solution that utilizes global data representations to mitigate noise. It transforms local data into privacy-preserving representations through dimensionality reduction and then applies a two-stage filtering process using k-nearest neighbor graphs to centrally aggregate clean data. Another approach focusing on filtering noisy samples is proposed in [30]. Alternative approaches introduce label correction techniques [31,32,33]. FOCUS [34] addresses the challenge of label noise in federated learning by using benchmark samples to assess the credibility of clients’ local data. FOCUS employs mutual cross-entropy to evaluate credibility and adjusts client weights accordingly through credit-weighted orchestration. Tested on synthetic and real-world datasets, FOCUS effectively reduces the impact of noisy labels and outperforms traditional FL methods. The authors in [35] propose a method that combines temporal-context contrastive learning with few-shot and self-supervised learning to extract fault data features from unlabeled datasets. This approach enables effective model training on small, label-deficient datasets, whereas [36] focuses on multi-organ CT segmentation using FL by incorporating knowledge distillation (KD) to mitigate catastrophic forgetting. This method involves training a multi-head U-Net model that leverages knowledge from a global model and pre-trained organ-specific models to improve segmentation accuracy across diverse datasets. The edge-model [37] enhances FL by using multiple global models to mitigate the impact of malicious users. Clients are randomly assigned to different global models during each training iteration, ensuring diverse input and comprehensive learning. By identifying and excluding malicious clients, the method improves the robustness of the global models. The final prediction for a test sample is determined by the global model with the highest accuracy, selected through a majority voting process among all global models.

Further, the Aorta framework, presented in [38], addresses the issue of label noise and device heterogeneity, simultaneously. It calibrates label noise by comparing model performance against a clean dataset and reconstructs clean data on each client using the global clean data available on the server. Clients are then selected for global aggregation based on the quality of their training data, ensuring that only those with high-quality data participate in the aggregation process. This selection is based on the quality of their training data, with low-quality clients being disregarded. In contrast, our approach diverges significantly from theirs. Instead of entirely neglecting low-quality clients, we include them in the aggregation process but apply penalties to their contributions. This ensures that the overall feature space is more comprehensively represented, maintaining the diversity and robustness of the global model while still managing the impact of low-quality data.

## 3. Background

### 3.1. Federated Learning

Federated learning (FL) is a decentralized approach to training machine learning models that enables edge devices to collaboratively train a global model while keeping their local data private. By allowing user devices to participate directly in the training process, FL leverages both the data diversity and computational resources of individual edge devices, potentially improving the effectiveness of the global model.

Although FL is related to distributed machine learning (ML) [39], there are significant differences. In distributed ML, the dataset is partitioned into smaller subsets, with each subset assigned to a computing node. These nodes may share data as needed, and the primary goal is to distribute the computational workload efficiently. In contrast, FL ensures that data remain on the local devices, focusing on preserving privacy by sharing only model updates between clients and the central server.

The typical FL framework consists of a central server coordinating a set of independent client devices. The training process follows a sequence of steps:

1. The central server initializes and distributes the global model to participating clients.
2. Each client trains the model locally using its private dataset and computes an updated version of the model.
3. The server aggregates the clients’ updates to refine the global model.

This iterative process aims to minimize an aggregated global loss function, defined as follows:

(1) $$f_{k}\left(\Theta^{k}\right)=\frac{1}{n_{k}}\sum_{i}^{N_{k}}l\left(x_{i},y_{i};\Theta^{k}\right)$$

(2) $$\min_{\Theta }f\left(\Theta \right)=\sum_{k=1}^{CxK}\frac{n_{k}}{n}f_{k}\left(\Theta^{k}\right)$$

where we have the following:

*x* is the data feature;
*y* is the data label;
$n_{k}$ is the local data size;
*n* is the total number of sample pairs;
*C* is the client participation ratio;
*l* is the loss function;
*k* is the client index.

Federated learning (FL) can be broadly categorized based on how data are distributed among clients, reflecting the structural relationships between datasets:

- **Horizontal Federated Learning (HFL)**: This paradigm applies when the datasets across different clients share the same set of features but consist of different samples. In other words, the data of each client correspond to a subset of the population, with identical feature spaces. For example, consider multiple hospitals collaborating to build a machine learning model for predicting disease risks. Each hospital collects data about patients using the same attributes, such as age, medical history, and lab results, but the patient populations do not overlap [40]. HFL is particularly effective in domains where institutions operate in similar contexts but are restricted from sharing sensitive data directly due to privacy concerns or regulations like GDPR.
- **Vertical Federated Learning (VFL)**: This paradigm applies when the datasets across different clients contain the same set of samples but differ in their features. VFL arises in situations where organizations possess complementary information about the same individuals or entities. For instance, a bank may hold transactional and financial data about its customers, while an e-commerce platform has data about their purchasing behavior. By collaboratively training a model without sharing raw data, these organizations can leverage their combined feature spaces to improve model performance [41]. VFL is especially valuable in cross-industry collaborations where the datasets are fragmented but can provide mutual benefits if integrated securely.
- **Federated Transfer Learning (FTL)**: In scenarios where datasets across clients differ in both features and samples, Federated Transfer Learning bridges the gap by leveraging transfer learning techniques. FTL enables knowledge sharing between domains with little or no overlap in data but with related tasks. For example, a healthcare provider in one region may have patient data with a rich set of features, while another region may have fewer features but a larger sample size. By transferring learned representations or knowledge, FTL allows both entities to enhance their models despite the dissimilarity in data distributions.

The flexibility of federated learning in accommodating diverse data distribution patterns makes it a versatile framework for collaborative training across various industries and applications. By categorizing data distribution scenarios into HFL, VFL, and FTL, FL ensures that organizations can choose strategies tailored to their specific privacy requirements and collaborative objectives. Moreover, this categorization underscores the adaptability of FL to heterogeneous environments while preserving its foundational principles of data privacy and decentralized model updates.

The capacity of FL to handle such diverse scenarios has made it a cornerstone technology for domains like healthcare, finance, and IoT, where privacy-preserving and collaborative learning are critical for building effective and ethical AI solutions.

### 3.2. The FedAvg Algorithm

The more diffused and known horizontal FL algorithm is FedAvg [42], proposed for the first time in [19]. FedAvg runs in parallel several steps of Stochastic Gradient Descent (SGD) on a small sampled subset of devices and then averages the obtained model updates via a central server once in a while. The main idea is that a central parameter server allows communication between the clients. This central node passes the global model to each client and collects the updated parameters from clients. This algorithm enables multiple devices to collaboratively train a machine learning model while keeping user data stored locally. The local models are aggregated into the global model, ensuring the fundamental requirements for data security and privacy protection. There are several studies that demonstrate how FedAvg accuracy decreases when it is applied to Non-IID and/or heterogeneous data [22].

This is due to the increasing divergence between the shared global model and the ideal model (based on IID data and heterogeneous data), which slows convergence and reduces overall performance. The detailed working of the FedAvg is shown in Algorithm 1.

The process begins with the server initializing the global model parameters and defining key configurations, such as the total number of clients, the fraction of clients to be selected in each round, the number of local training epochs, and the local batch size. Once initialized, the training proceeds over multiple iterative rounds.

At the start of each round, the server selects a subset of clients, determined by the specified fraction of clients to participate in that round. These selected clients receive the current version of the global model, which the server broadcasts to them. Each client then initializes its local model with the global parameters and performs training on its own private dataset. This local training involves updating the model parameters using a method like Stochastic Gradient Descent for a fixed number of epochs. Once the training is complete, the clients send their updated model parameters back to the server, along with information about the size of their local dataset.
**Algorithm 1** Federated averaging (FedAvg) algorithm.**Require:** *K*: Total number of clients. *F*: Fraction of clients selected in each round. *E*: Number of local training epochs. *B*: Local batch size. $w_{0}$: Initial global model parameters. **Ensure:** Updated global model parameters $w_{T}$ after *T* rounds.
  1:**Initialize:** Set the initial global model parameters $w_{0}$. Initialize client set S={1,2,…,K}.  2:**for** each round t=1,2,…,T **do**  3:    **Server Selection Phase:**  4:    Randomly sample a fraction F×K clients from the client set S.  5:    Broadcast the current global model parameters $w_{t}$ to the selected clients.  6:    **Client Update Phase (Executed by each selected client):**  7:    **for** each selected client *k* **do**  8:        Initialize local model with global parameters $w_{t}$.  9:        Perform local training on the client’s own data for *E* epochs using the update rule:$$w_{k}^{t+1}=w_{k}^{t}-\eta \nabla L_{k}\left(w_{k}^{t}\right)$$
where η is the learning rate, and $L_{k}$ is the local loss function of client *k*.  10:        Return updated local model parameters $w_{k}^{t+1}$ and the number of local data points $n_{k}$ to the server.  11:    **end for**  12:    **Server Aggregation Phase:**  13:    The server collects the local model updates $w_{k}^{t+1}$ and corresponding data sizes $n_{k}$.  14:    Aggregate the local updates using a weighted average:$$w_{t+1}=\frac{\sum_{k=1}^{m}n_{k}\cdot w_{k}^{t+1}}{\sum_{k=1}^{m}n_{k}}$$
where *m* is the number of selected clients in the round.  15:    **Update Global Model:** Update the global model with $w_{t+1}$.  16:**end for**  17:**return:** After *T* rounds, return the final global model parameters $w_{T}$.


The server collects these updates from the participating clients and aggregates them to update the global model. The aggregation is performed using a weighted averaging method, where the contribution of each client’s update is proportional to the size of its local dataset. This ensures that clients with larger datasets have a greater influence on the updated global model.

This process of selecting clients, training locally, and aggregating updates is repeated over multiple rounds. By the end of the specified number of rounds, the server produces a final global model that has been trained collaboratively across all clients while ensuring that the client’s raw data never leave their devices. The FedAvg algorithm is particularly effective because it balances privacy, efficiency, and the ability to leverage data from diverse and distributed sources. It reduces communication costs by allowing clients to perform multiple local updates before sending their results to the server, making it well suited for real-world federated learning scenarios.

## 4. The DQFed Approach

**Figure 1:**
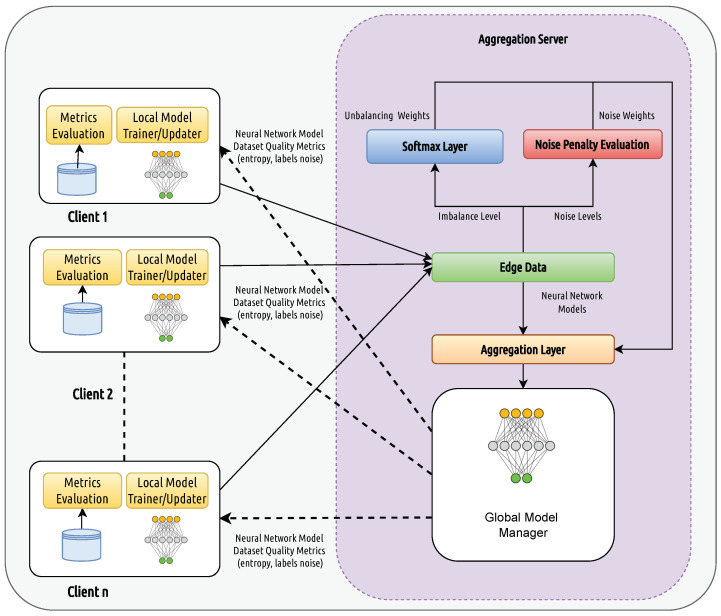
The DQFed architecture.

The DQFed approach extends the FedAvg algorithm by replacing the weights average with a quality-driven aggregation. This aims to obtain a more robust model from peripheral clients. It ensures the identification of noisy and imbalanced data and activates optimization strategies to improve the model accuracy. The DQFed architecture is described in Figure 1. The clients ($Client_{1},..Client_{K}$), represented at the left side of the figure, collect locally the values of the monitored parameters that are used to train the local neural networks. At the local level, a metric computation is also performed to evaluate the quality of the local dataset. The trained models are then sent to the Edge Data component within the values of the evaluated metrics. The Edge Data component of the aggregation server aggregates the local models on the basis of their corresponding metrics.

The metrics received by the Edge Data are then aggregated through an aggregator with the aim to obtain an evaluation of the imbalance and noise level of each considered node. The Softmax Layer component of the aggregation server receives from the Edge Data the imbalance vector of the imbalance metric value (imbalance level) and computes an imbalance probability vector (imbalance weight).

Each element *z* of the vector has a probability $pu_{i}$ that the input belongs to $class_{i}$ according to the following formula:

(3) $$pu_{i}=\frac{e^{z_{i}}}{\Sigma_{j=1}^{N}e^{z_{j}}}$$

where we have the following:

*e* is the base of the natural logarithm;
*N* represents the number of classes;
$\Sigma_{j=1}^{N}e^{z_{j}}$ is the sum of the exponentials of all elements in *z*

The Softmax Layer computes, on the base of the received metrics, the imbalance weights assigned to the models of different clients during the aggregation phase according to their imbalance level and ensures that the sum of weights is equal to 1.

Similarly, in the context of the aggregation server, the Noise Penalty Evaluation component receives the vectors of the noise metric values (estimating the noise levels for each client) and computes the noise weights to modulate the aggregation of models.

The Noise Penalty Evaluation component computes, on the base of the received metrics, the noise weights assigned to the models of different clients during the aggregation phase according to their noise level and ensures that the sum of weights is equal to one.

All the vectors of weights are then sent to the Aggregation Layer. This component combines the parameters of the client models by weighting them according to the weights derived from the Noise Penalty Evaluation and Softmax Layer, and generates the global model. The generated global model is sent to the Global Model Manager who is in charge of distributing it to the local clients. In this way, the local networks are updated, and the evolution and the improvement of the DQFed is ensured. This study proposes for local and global models the adoption of the CNN networks [43,44]. However, the proposed approach is agnostic with respect to the model adopted at the local and global levels.

The proposed DQFed has been developed using an FL framework called Flower (https://flower.ai, accessed on 8 May 2025).

### 4.1. The Quality Model

DQFed is an approach to integrate several quality metrics according to the type of context and the planned quality goals. In this study, we propose a quality model aimed at reducing the local noise level and imbalance. In particular, the adopted quality model evaluates the adoption of the Shannon entropy (SE) quality metric that gives information about the entropy level of the data and of the noise level evaluated by a variational autoencoder (VAE).

#### 4.1.1. The Shannon Entropy

Claude Shannon introduced the Shannon entropy metric [45] to quantify the amount of information or uncertainty associated with a system according to the information theory.

Shannon entropy (*SE*) is defined by the following formula:

(4) $$SE=-\Sigma_{i=1}^{N}\;log\frac{c_{i}}{V}$$

where we have the following:

*V* is the number of values in a dataset;
*N* represents classes;
$c_{i}$ represents the size of class *i*.

*SE* can be used, in classification tasks, to evaluate the level of uncertainty or unpredictability in a dataset. However, considering a classification dataset composed of different classes, *SE* can be useful to evaluate the degree of diversity in the class distribution. When a dataset is balanced (i.e., there is the same number of instances for each class), the entropy reaches its maximum (greatest level of uncertainty or diversity in class distribution). Conversely, in cases of extreme class imbalance (e.g., when one class dominates the dataset), entropy decreases, signaling reduced diversity in the class distribution.

In the proposed approach, we apply *SE* to quantify the diversity present in the data collected at the local level and apply strategies aimed at minimizing data diversity at the global level.

#### 4.1.2. Noise Detection and Penalization Score

Noise detection employs a supervised variational autoencoder to detect noise in the data contributed by clients in a federated learning system. Noise detection involves training the supervised variational autoencoder to reconstruct data and identify anomalies or inconsistencies that deviate from the expected patterns, which indicate noise. Once noise is detected, a penalization score is calculated for each client based on the level of noise in their contributions. These penalization scores are then used to adjust the aggregation process, reducing the weight of noisy clients in the global model update. This two-step process ensures that the global model is less affected by unreliable data, improving overall performance and robustness.

**Figure 2:**
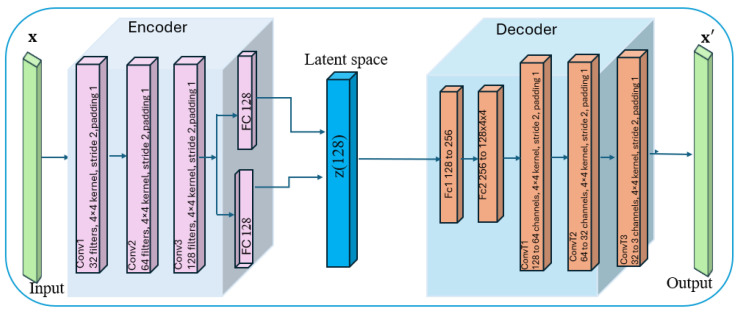
Architecture of the variational autoencoder model used for noise detection.

We employed a supervised variational autoencoder (VAE) model to detect noise within the local datasets, as outlined in Algorithm 2. The architecture of the VAE is illustrated in Figure 2, and a comprehensive summary of all relevant hyperparameters is provided in Table 1 to provide a detailed overview of the model configuration.
**Algorithm 2** VAE training for noise detection.**Require:** Training dataset *D*, number of epochs *E*, batch size *B*, learning rate α, noise rate η **Ensure:** Trained VAE model *M*
  1:Initialize VAE model *M* with input dimension $d_{in}$, hidden dimension $d_{h}$, latent dimension $d_{l}$, and number of classes *N*  2:Initialize Adam optimizer *O* with learning rate α  3:$D_{noisy}\leftarrow$ AddLabelNoise(D,η)  4:**for** epoch=1 to *E* **do**  5:    $L_{total}\leftarrow 0$  6:    **for** each batch (X,Y) in $D_{noisy}$ with size *B* **do**  7:        X← Reshape$\left(X,\left(B,d_{in}\right)\right)$  8:        Y← ConvertToLongTensor(Y)  9:        $\left(\hat{X},\mu ,\log\sigma^{2},logits\right)\leftarrow M\left(X\right)$  10:        $L_{recon}\leftarrow$ BCELoss$\left(\hat{X},X\right)$  11:        $L_{class}\leftarrow$ CrossEntropyLoss$\left(\hat{Y},Y\right)$  12:        $L_{KL}\leftarrow -0.5\sum \left(1+\log\sigma^{2}-\mu^{2}-e^{\log\sigma^{2}}\right)$  13:        $L\leftarrow L_{recon}+L_{KL}+L_{class}$  14:        BackwardPass(L)  15:        UpdateParameters(O)  16:        $L_{total}\leftarrow L_{total}+L$  17:    **end for**  18:    **if** epochmod10=0 **then**  19:        $S_{noisy}\leftarrow$ DetectLabelNoise$\left(M,D_{noisy}\right)$  20:    **end if**  21:**end for**  22:**return** *M*


The VAE algorithm operates through two primary mechanisms: noise recognition and noise detection, which involve the metrics of reconstruction loss (Lrecon) and KL divergence (LKL). The model is trained using the Adam optimizer with a learning rate of 1 $\times \;10^{-3}$, monitoring both total loss and reconstruction loss to gauge the model’s learning progress. The first mechanism encompasses the model architecture, forward pass, loss function, and training procedure. The model architecture includes an encoder, a decoder, and a classifier, all of which are implemented using fully connected neural networks. Further, the forward pass involves encoding, decoding, and classification based on the model architecture discussed. Finally, the loss function combines three components: (a) Reconstruction Loss $L_{recon}:\;L_{recon}\leftarrow$ BCELoss$\left(\hat{X},X\right)$, where BCELoss is the Binary Cross-Entropy loss, (b) KL Divergence $\left(L_{KL}\right):\;L_{KL}\leftarrow -0.5\sum \left(1+\log\sigma^{2}-\mu^{2}-e^{\log\sigma^{2}}\right)$, which is a term that encourages the learned latent distribution to be close to the prior N(0,I), and (c) Classification Loss$\left(L_{class}\right):\;L_{class}\leftarrow$ CrossEntropyLoss$\left(\hat{Y},Y\right)$, where *Y* is the true label and $\hat{Y}$ shows predicted class probabilities. Lastly, the model is trained using the Adam optimizer reference with a learning rate of 1 $\times \;10^{-3}$. This training monitors both the total loss and the reconstruction loss, providing insights into the model’s learning progress. Once the variational autoencoder (VAE) is trained, a noise detection mechanism is applied to identify potentially mislabeled samples based on reconstruction errors. The process involves generating predictions and confidence scores for each sample. If the predicted label differs from the given label and the confidence score exceeds a defined threshold, the sample is marked as potentially noisy. This threshold is determined using the statistical characteristics of the reconstruction errors, specifically calculated from their mean and standard deviation to ensure robust noise detection criteria.

#### 4.1.3. Penalization Strategies for Noise and Imbalance

Once noisy labels are detected within the datasets, we employ a penalization algorithm to reduce their impact, referred to as Noise Reduction Penalization (NRP). This algorithm adjusts each client’s contribution in the federated learning setting based on the noisy samples in their data. The NRP score for each client is calculated, considering the total number of clients (K) and samples (S). After penalization, these NRP scores and entropy are used in an aggregation algorithm to combine client contributions and evaluate the model’s overall performance, ensuring robustness against introduced noise and mislabeling. The NRP calculation and DQFed Aggregation are described in Algorithm 3.
**Algorithm 3** NRP score calculation and DQFed Aggregation.**Require:**  1:K: Set of clients  2:$S_{k}$: Number of samples provided by client *k*, ∀k∈K  3:$n_{noisy,k}$: Number of noisy samples provided by client *k*, ∀k∈K  4:$n_{noisy}$: Total number of noisy samples detected in FL  5:*S*: Total number of samples in federated learning  6:NRP score = $p_{k}$**Ensure:** NRP score = $p_{k}$
  7:Initialize penalties: $p_{k}$←{}  8:Compute Overall Proportion of Noisy Samples:        $P_{noisy}\leftarrow \frac{n_{noisy}}{S}$  9:Compute the Portion of individual Noise: $P_{noisy,k}\leftarrow \frac{n_{noisy,k}}{S_{k}}$  10:**for** each client *k* **do**  11:    Compute the Penalty for the client:  12:    $p_{k}\leftarrow \left(\frac{P_{\mathrm{noisy},k}}{P_{\mathrm{noisy}}}\right)\times \left(\frac{S_{k}}{S}\right)\times \log\left(1+S_{k}\right)$  13:**end for**  14:DQFed Aggregation Phase:  15:The server collects the local model updates $w_{k}^{t+1}$ and corresponding data sizes $n_{k}$.  16:Aggregate the local updates using a weighted average based on entropy and NRP score:$${\left(w_{t+1}^{\mathrm{entropy}}\right)}_{k}=\frac{n_{k}\cdot e_{k}\cdot w_{k}^{t+1}}{\sum_{j=1}^{m}n_{j}\cdot e_{j}}$$$$w_{t+1}^{\mathrm{final}}=\frac{\sum_{k=1}^{m}n_{k}\cdot p_{k}\cdot {\left(w_{t+1}^{\mathrm{entropy}}\right)}_{k}}{\sum_{k=1}^{m}n_{k}\cdot p_{k}}$$
In the first aggregation step, $w_{t+1}^{\mathrm{entropy}}$ is computed as a weighted average of all clients’ local updates weighted by their data sizes and entropy scores.In the second aggregation, $w_{t+1}^{\mathrm{final}}$ effectively applies the NRP scores as secondary weights to the already entropy-weighted updates.
       where *m* is the number of selected clients in the round.  17:Update Global Model: Update the global model with $w_{t+1}^{\mathrm{final}}$.  18:**return:** the final global model parameters.


## 5. Empirical Validation

The empirical validation of the proposed approach is aimed at answering the research questions (RQ1, RQ2, RQ3, and RQ4) proposed in the introduction. The datasets adopted in the validation are obtained starting from the well-known open-source CIFAR-10 dataset (https://www.cs.toronto.edu/~kriz/cifar.html). This dataset comprises 60,000 32 × 32 color images divided into 10 classes, with each class containing 6000 images. From the original dataset, we created *n* datasets $\left(D_{1},\ldots ,D_{n}\right)$ by introducing imbalance and injecting noise into them. Each dataset maintains the standard CIFAR class structure (ten classes). In the next subsections, the datasets unbalancing and noise injection methods are described.

### 5.1. Datasets and Imbalance Injection

**Figure 3:**
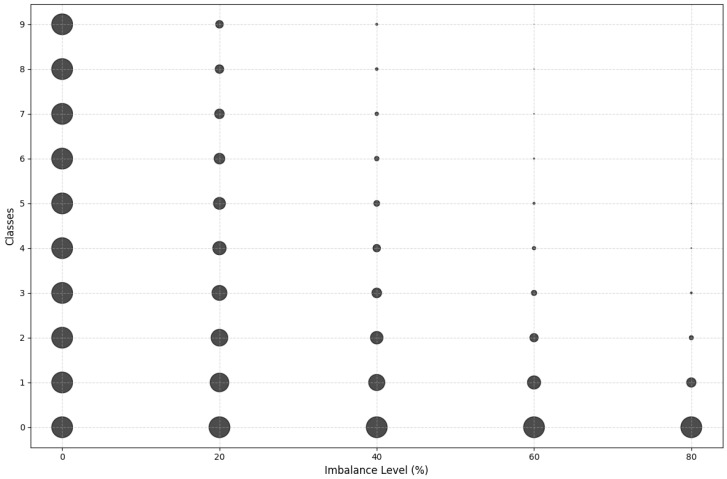
Example of the classes distribution across imbalance levels.

In the way to answer research question RQ1, the imbalance is introduced in the datasets associated with the n clients [$C_{1}$, …,$C_{k}$,…,$C_{n}$] by using augmentation and transformation techniques. The dataset CIFAR-10 is initially split into n datasets for *n* clients. For the assessment, we repeat the experiment for n ranging from n = 5 to n = 25 for different levels of imbalance. For instance, Figure 3 reports the bubble plot of class distribution across different imbalance levels.

Entropy starts high at an imbalance ratio of 0 (balanced classes) and gradually decreases as the imbalance ratio increases due to higher skewness in class distributions.

### 5.2. Datasets and Noise Injection

The imbalanced datasets are further modified by injecting noise into them. The noise injection is performed using the noise injection. This means that the true labels of images are replaced by random labels of other classes, switching label samples according to the reference noise levels.

Algorithm 3 calculates the NRP score for each client. Based on these scores, we aggregate each client’s contributions using the DQfed strategy as described in our previous work [15]. This aggregation allows us to evaluate the model’s performance effectively.

### 5.3. The Experiment Setting

Different experiments are proposed to answer the research questions RQ1, RQ2, RQ3, and RQ4. We perform an ablation-style study on RQ1 and RQ2 to discuss the individual impacts of imbalance and noise. The robustness of the DQFed is evaluated by introducing the F1-score in both cases. It is computed as the harmonic mean between precision and recall:

(5) $$F1-Score=2\cdot \frac{Precision\cdot Recall}{Precision+Recall}.$$

The DQFed and some alternative baseline approaches are used for each dataset including different imbalance and mislabeling levels. Different combinations of imbalance and mislabeling levels are considered to evaluate the robustness of the proposed approach when there is a combination of imbalance and mislabeling. The FL strategy models considered in this study are FedAvg, FedAvgM, FedProx, and FedOpt.

Question RQ3 aims to evaluate the robustness of the proposed approach when the FL architecture changes. This study, in particular, considers FL models characterized by different numbers of clients (15, 20, and 25). The DQFed robustness for all the considered clients is evaluated using the described F1-score, and a comparison with the alternative FL strategies is also performed. Finally, RQ4 aims to evaluate the effectiveness of the DQFed approach compared with the other strategy models. According to this, the execution time for the considered models is computed for all the considered FL architectures. The experiments are performed on a server with an Intel Core i7 10th generation, 16 GB of RAM, and NVIDIA GeForce Experience version 3.27 GPU.

## 6. Results and Discussion

In this section, the results obtained for each research question are reported and discussed.

### 6.1. Results and Discussion for RQ1

**Figure 4:**
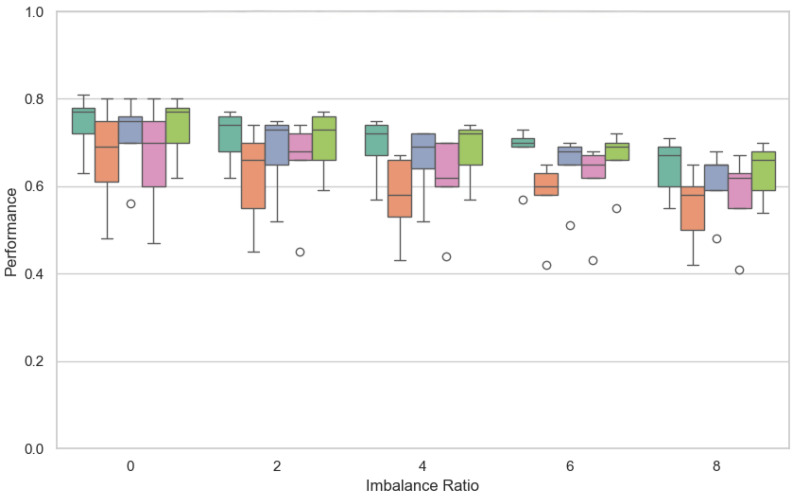
Performance (F1-score) of each FL strategy with respect to the imbalance (25 clients).

Figure 4 shows the F1-score distribution of the proposed approach and all the alternative strategies for different values of the imbalance ratio (0, 2, 4, 6, 8).

Even though several FL architectures are considered in this study, we briefly report the data distribution when the number of clients is 25 in this section. The figure shows that when there is an imbalance (the imbalance ratio is equal to zero), all the strategies are quite robust. In this case, the best values are obtained by DQFed and FedOpt. When the imbalance ratio starts to increase, the robustness decreases in any case. However, the figure shows that DQFed and FedOpt are also always more robust than the other approaches. Comparing DQFed and FedOpt distributions, we can observe that DQFed is always more robust than FedOpt, even if not always in a significant way. Greater distribution for DQFed is obtained when the imbalance ratio is increased. Table 2 confirms the above considerations showing the best values of F1-score for all the considered strategies.

### 6.2. Results and Discussion for RQ2

**Figure 5:**
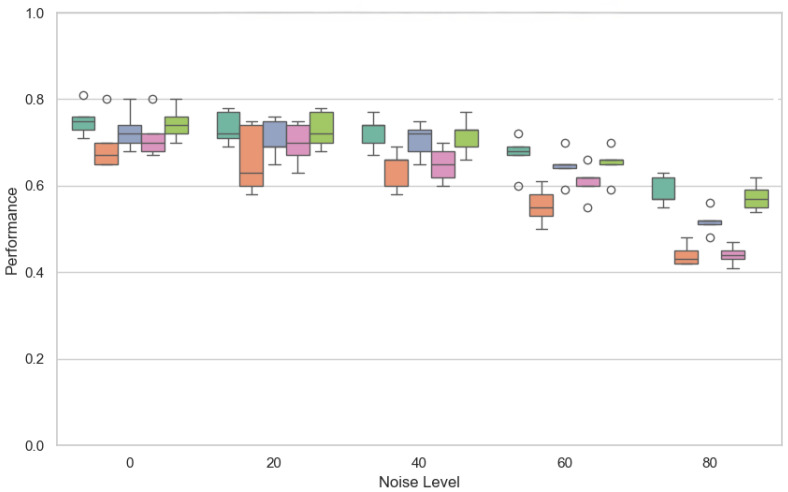
Performance (F1-score) of the explored FL strategies with respect to the noise level (25 clients).

Figure 5 shows the distribution of the F1-score for different noise levels when the number of clients is 25.

When the noise is zero, similar performance is obtained by DQFed and FedOpt strategies (they are the more robust strategies among the considered approaches). When the noise level increases, the DQFed strategy shows the best values of the F1-score. In particular, when the percentage of the noise level is 60, there is a significant improvement of DQFed with respect to all the alternative approaches. To be more explicit, we observe the following:

- **Entropy-based weighting**, as shown in Table 2, provides a relative improvement in F1-score over FedAvg under high imbalance conditions, achieving notably higher performance (0.71 compared to 0.65 at the highest imbalance level).
- **Noise-based weighting**, as demonstrated in Table 3, results in a substantial relative improvement in F1-score over FedAvg under high noise conditions, with performance increasing significantly (0.63 compared to 0.48 at 80% noise).

**Figure 6:**
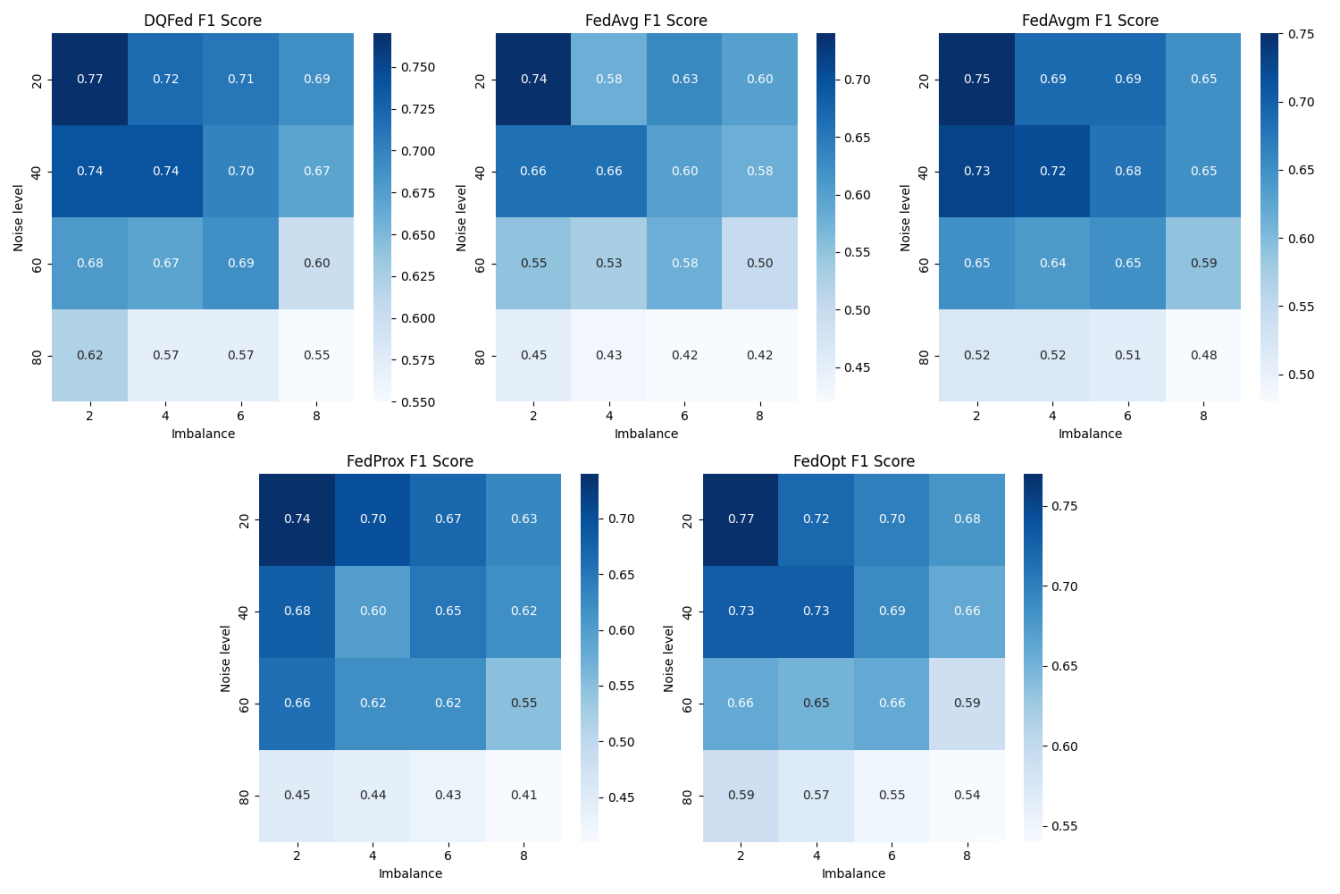
F1-score of the explored FL strategies with respect to noise and imbalance.

The robustness of all the considered approaches is also evaluated when there is a combination of imbalance and noise at different levels of severity. Figure 6 reports the F1-score of studied strategies with respect to increasing levels of noise and imbalance. DQFed confirms the best strategy concerning the presence of both high levels of noise and imbalance. This can be seen in the figure where it outperforms all the considered SOTA methods, being the only strategy with an F1-score better than 0.6 when the noise level is around 80%.

### 6.3. Results and Discussion for RQ3

Question RQ3 aims to investigate the robustness of DQFed when the number of clients changes (it means to evaluate the robustness of the approach when different FL architectures are considered).

**Figure 7:**
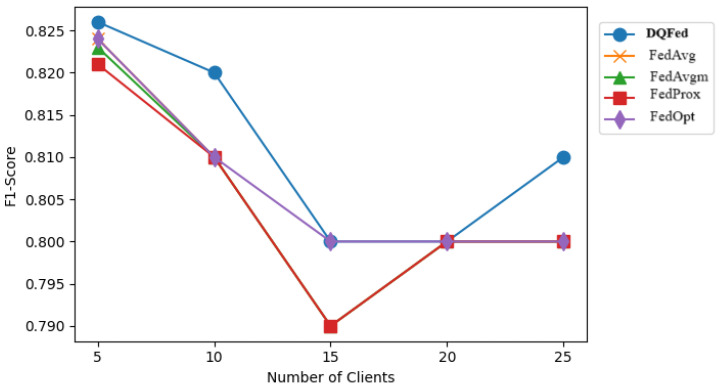
F1-score of the explored FL strategies with respect to clients at 0% noise.

**Figure 8:**
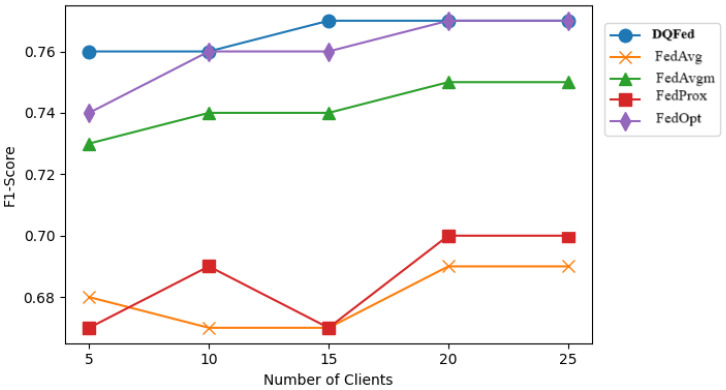
F1-score of the explored FL strategies with respect to clients at 40% noise.

**Figure 9:**
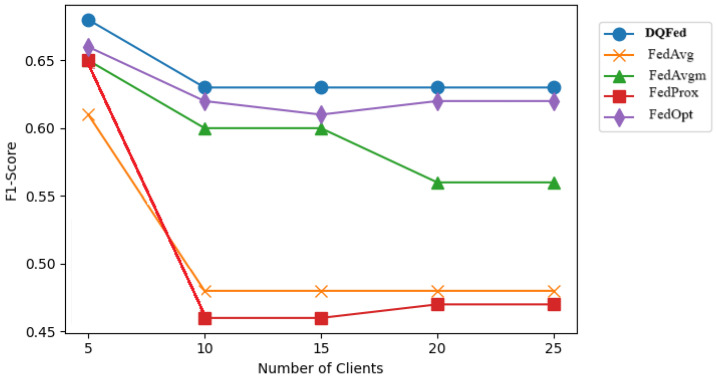
F1-score of the explored FL strategies with respect to clients at 80% noise.

**Figure 10:**
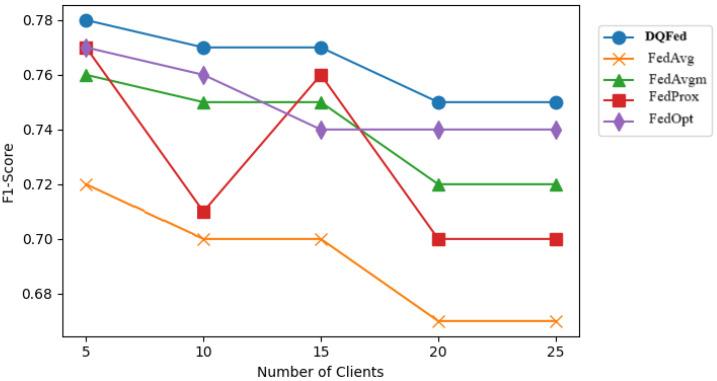
Performance (F1-score) of the explored FL strategies with respect to clients at 0-10 ratio of imbalance.

**Figure 11:**
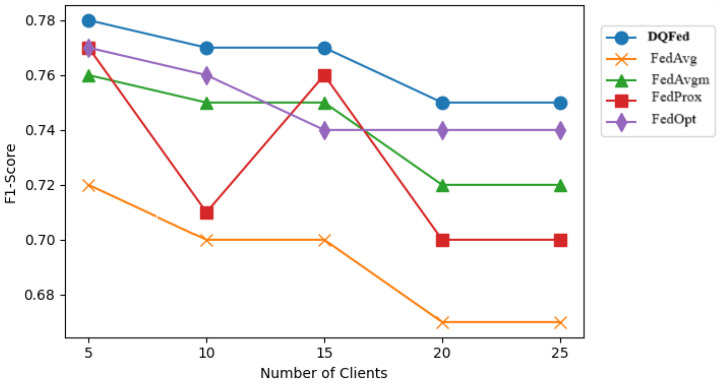
Performance (F1-score) of the explored FL strategies with respect to clients at 4-10 ratio of imbalance.

**Figure 12:**
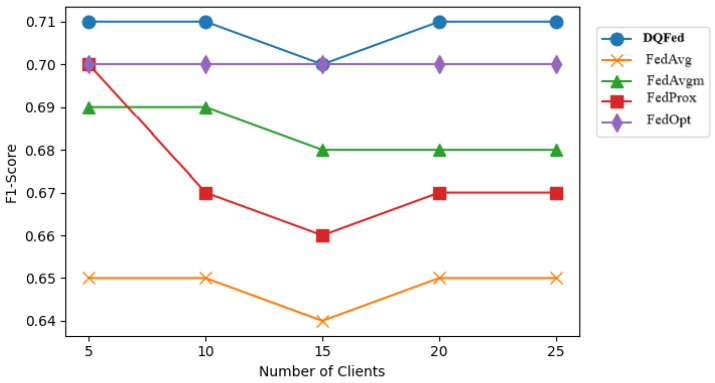
Performance (F1-score) of the explored FL strategies with respect to clients at 8-10 ratio of imbalance.

Figure 7, Figure 8, and Figure 9 report respectively the F1-score obtained when the percentage of noise level is 0, 40, and 80 for different number of clients. Figure 10, Figure 11 and Figure 12 show the obtained F1-score with respect to the number of clients and imbalance level of (0–10, 4–10, 8–10), respectively. In all the cases, we can observe that the number of clients influences the F1-score. Generally, the performance of the model decreases when the number of clients increases. However, in all the cases, the DQFed gives the best performance (almost equal) with respect to the other approaches.

### 6.4. Results and Discussion for RQ4

**Figure 13:**
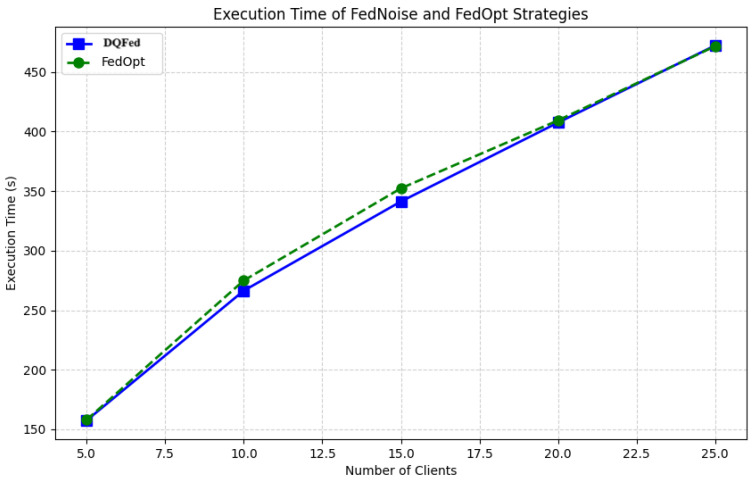
Execution time for DQFed and FedOpt.

Figure 13 reports the execution time for DQFed and FedOpt when the number of clients changes. The figure shows very similar execution times. However, when the numbers of clients are 10 and 15, the proposed approach shows a reduced execution time.

### 6.5. Wilcoxon Signed-Rank Test

To rigorously evaluate the statistical significance of the observed improvements achieved by our proposed DQFed method over baseline federated learning approaches (FedAvg, FedAvgm, FedProx, and FedOpt), we conduct a Wilcoxon signed-rank test. This non-parametric statistical test is selected because it does not assume a normal distribution of performance scores, making it particularly suitable for small-sample paired comparison settings typical in federated learning evaluations. Table 4 reports the *p*-values obtained when comparing DQFed to each baseline method with respect to Research Question 1 (RQ1). The results consistently demonstrate statistically significant improvements (p<0.05) across all comparisons. Notably, the *p*-value for DQFed versus FedAvg is particularly low (p=0.0025), highlighting a strong difference in performance favoring DQFed. These outcomes provide robust evidence that DQFed achieves significant gains over standard federated learning algorithms under the experimental settings addressing RQ1.

Similarly, Table 5 presents the statistical comparison outcomes for Research Question 2 (RQ2). Again, DQFed significantly outperforms each of the compared baselines with all *p*-values falling below the significance threshold of α=0.05. The consistency of these findings across multiple baseline methods strengthens the claim that DQFed offers generalizable performance improvements, even under the different experimental conditions considered for RQ2. This statistical validation underlines the effectiveness and robustness of our proposed approach across different federated learning settings.

### 6.6. Convergence Rate over Rounds

**Figure 14:**
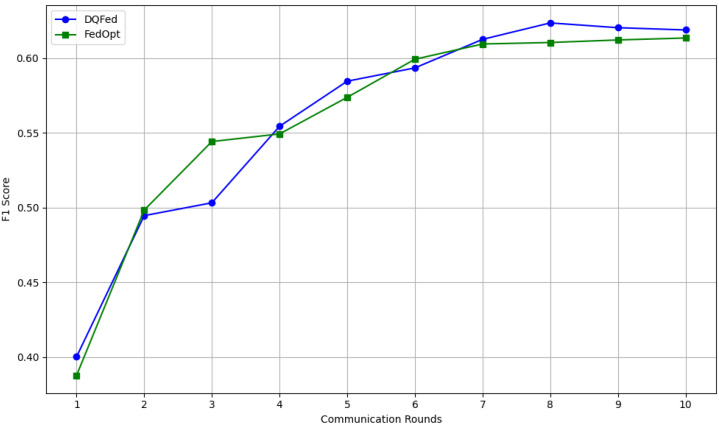
Convergence rate for DQFed and FedOpt.

In order to analyze convergence and stability behavior, we compare the F1-scores of Strategy DQFed and FedOpt(baseline) over 10 communication rounds. We observe that Strategy DQFed consistently maintains competitive or superior performance across rounds, with smoother progression and no signs of instability or oscillation. For further illustration, we include a convergence graph (Figure 14) showing the round-wise F1-scores progression for both strategies. This indicates that our penalization mechanism not only improves local quality but also maintains stable global convergence.

## 7. Conclusions

This paper introduces DQFed, a quality-driven FL approach designed to address key challenges in distributed machine learning, including data heterogeneity (Non-IID distributions) and noise heterogeneity (mislabeling and inconsistencies). An empirical evaluation of the proposed DQFed method is conducted on 25 datasets derived from the CIFAR-10 dataset. The evaluation results show that DQFed improves model accuracy and reliability by tackling class imbalance and noisy data, demonstrating superior results compared to existing FL frameworks. The evaluation also shows that the number of clients influences the F1-score of the explored FL strategies, but in all the cases, the proposed approach gives the best (or equal) score. Finally, the execution times for DQFed and FedOpt are very similar. This makes it a promising solution for robust FL applications in privacy-sensitive and data-diverse fields like healthcare and IoT.

## Figures and Tables

**Table 1:** Training hyperparameters for the VAE model.

Hyperparameter	Description
Optimization Algorithm	Adam optimizer
Learning Rate	0.001
Batch Size	128
Training Epochs	16
Loss Function	Binary Cross-Entropy + KL-Divergence
Device	CUDA (if available), otherwise CPU

**Table 2:** Performance (F1-score) of each FL strategy with respect to the imbalance (25 clients).

Clients	Imbalance	Dqfed	FedAvg	FedAvgm	FedProx	FedOpt
25	0	0.81	0.8	0.8	0.8	0.8
25	2	0.76	0.7	0.74	0.72	0.76
25	4	0.75	0.67	0.72	0.7	0.74
25	6	0.73	0.65	0.7	0.68	0.72
25	8	0.71	0.65	0.68	0.67	0.7

**Table 3:** Performance (F1-score) of each FL strategy with respect to the noise (25 clients).

Clients	Noise %	DQFed	FedAvg	FedAvgm	FedProx	FedOpt
25	0	0.81	0.8	0.8	0.8	0.8
25	20	0.78	0.75	0.76	0.75	0.78
25	40	0.77	0.69	0.75	0.7	0.77
25	60	0.72	0.61	0.7	0.6	0.7
25	80	0.63	0.48	0.56	0.47	0.62

**Table 4:** Statistical significance test of DQFed compared to other methods addressing RQ1.

Comparison	*p*-Value	Significant (α=0.05)?
DQFed vs. FedAvg	0.0025	Yes
DQFed vs. FedAvgm	0.041	Yes
DQFed vs. FedProx	0.034	Yes
DQFed vs. FedOpt	0.046	Yes

**Table 5:** Statistical significance test for of DQFed compared to other methods addressing RQ2.

Comparison	*p*-Value	Significant (α=0.05)?
DQFed vs. FedAvg	0.034	Yes
DQFed vs. FedAvgm	0.038	Yes
DQFed vs. FedProx	0.041	Yes
DQFed vs. FedOpt	0.038	Yes

## Data Availability

The dataset is available at https://www.cs.toronto.edu/~kriz/cifar.html 10 January 2024.
